# Aquaporin 1, 3, and 5 Patterns in Salivary Gland Mucoepidermoid Carcinoma: Expression in Surgical Specimens and an In Vitro Pilot Study

**DOI:** 10.3390/ijms21041287

**Published:** 2020-02-14

**Authors:** Mérin Barbara Stamboni, Ágatha Nagli de Mello Gomes, Milena Monteiro de Souza, Katia Klug Oliveira, Claudia Fabiana Joca Arruda, Fernanda de Paula, Barbara Beltrame Bettim, Márcia Martins Marques, Luiz Paulo Kowalski, Clóvis Antônio Lopes Pinto, Victor Elias Arana-Chavez, Silvia Vanessa Lourenço, Cláudia Malheiros Coutinho-Camillo

**Affiliations:** 1 International Research Center, A.C.Camargo Cancer Center, Rua Taguá 440, São Paulo 01508-010, Brazil; 2 National Institute of Science and Technology in Oncogenomics and Therapeutic Innovation, São Paulo 05403-010, SP, Brazil; 3 Department of General Pathology, Dental School, University of São Paulo, São Paulo 05508-000, Brazil; 4 Department of Restorative Dentistry, School of Dentistry, University of São Paulo, São Paulo 05508-000, Brazil; 5 Department of Head and Neck Surgery and Otorhinolaryngology, A.C.Camargo Cancer Center, São Paulo 01525-001, Brazil; 6 Department of Anatomic Pathology, A.C.Camargo Cancer Center, São Paulo 01525-001, Brazil; 7 Laboratory of Oral Biology, Department of Biomaterials and Oral Biology, Dental School, University of São Paulo, São Paulo 05508-000, Brazil

**Keywords:** aquaporins, mucoepidermoid carcinoma, immunohistochemistry, immunofluorescence, electron microscopy

## Abstract

Salivary gland aquaporins (AQPs) are essential for the control of saliva production and maintenance of glandular structure. However, little is known of their role in salivary gland neoplasia. Salivary gland tumors comprise a heterogeneous group of lesions, featuring variable histological characteristics and diverse clinical behaviors. Mucoepidermoid carcinoma (MEC) is the most common salivary gland malignancy. The aim of this study was to evaluate the expression of AQP1, AQP3, and AQP5 in 24 MEC samples by immunohistochemistry. AQP1 expression was observed in vascular endothelium throughout the tumor stroma. AQP3 was expressed in epidermoid and mucosal cells and AQP5 was expressed in mucosal cells of MEC. These proteins were expressed in the human MEC cell line UH-HMC-3A. Cellular ultrastructural aspects were analyzed by electron microscopy to certificate the tumor cell phenotype. In summary, our results show that, despite the fact that these molecules are important for salivary gland physiology, they may not play a distinct role in tumorigenesis in MEC. Additionally, the in vitro model may offer new possibilities to further investigate mechanisms of these molecules in tumor biology and their real significance in prognosis and possible target therapies.

## 1. Introduction

Aquaporins (AQPs) are integral membrane proteins that form channels that facilitate water and solute transport [1,2,3,4]. Due to their role as water channel structures, they are essential in the physiology of salivary glands and saliva production [5,6]. Several studies have suggested that AQPs may be involved in processes such as cellular migration, angiogenesis, organ regeneration, wound healing, and tumorigenesis [2,4,6,7,8,9,10,11,12].

Altered expression of AQPs has been observed in multiple tumors. Some authors have associated AQP expression with tumor grade, angiogenesis, and tumor-associated edema. However, the molecular mechanisms of AQP function in tumors are unclear because both overexpression and underexpression of these proteins have been related to poor prognoses of adenocarcinomas of many organs such as lung, pancreas, stomach, liver, and colon, among others [2,8,9,10,11,12,13,14,15,16,17,18]. A few studies have also evaluated the expression of AQPs in the development and progression of salivary gland tumors, with special attention given to AQP3 and AQP5, because they are the predominant AQPs of normal salivary gland tissue. These studies were primarily descriptive and did not explore potential associations between AQPs and clinical outcomes or histopathology [15,16].

Mucoepidermoid carcinoma (MEC) is the most common malignancy of the salivary gland. Histologic grade and anatomic site have prognostic and therapeutic relevance, and metastasis is an indicator of poor prognosis [19,20,21,22,23]. Thus, the identification of an expression profile of AQPs in MEC and its association with clinical and pathological features could not only provide a better understanding of the molecular basis of these lesions but might also identify prognostic biomarkers. The aim of our study was to investigate the expression of AQP1, AQP3, and AQP5 in MEC and to associate their expression with patient demographic, clinical, and pathological characteristics.

## 2. Results

**Figure 1:**
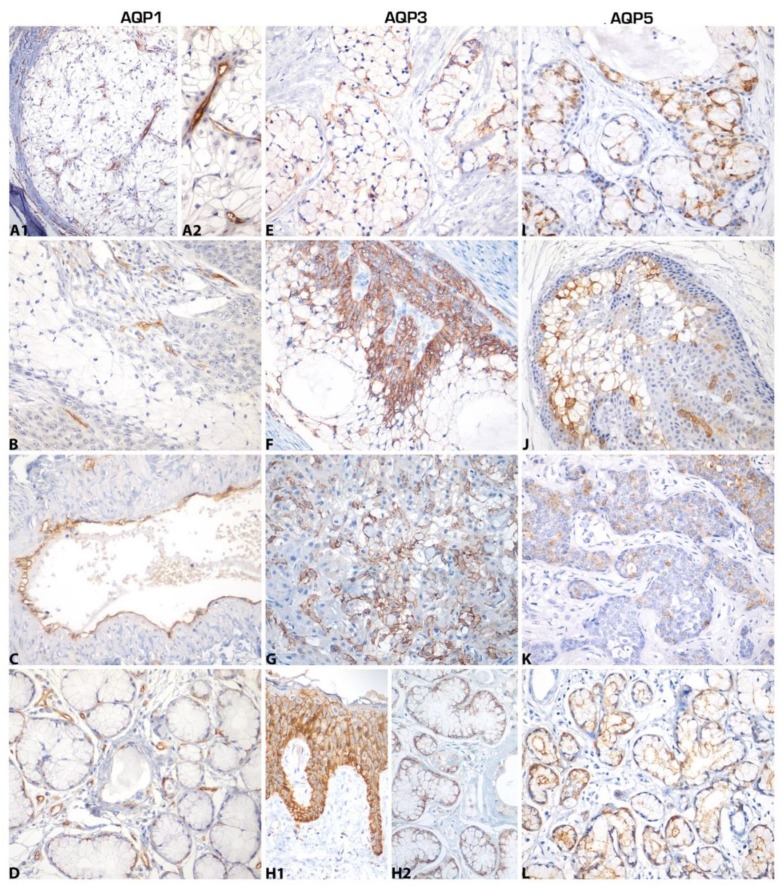
Expression of aquaporins (AQPs) in mucoepidermoid carcinoma (MEC). (**A**,**B**,**C**) AQP1 expression was observed in tumor vascular endothelium and was absent in tumor parenchyma; (**A1**) AQP1 expression in tumor vasculature; (**A2**) AQP1 expression in higher magnification. (**D**) Normal salivary gland expressed AQP1 in vasculature. (**E**,**F**,**G**) AQP3 membrane expression in mucous, intermediate, and epidermoid neoplastic cells. (**H1**) AQP3 expression in all layers of normal oral mucosal epithelium. (**H2**) Expression in acinar mucous cells of normal salivary gland and some ductal cells. (**I**,**J**,**K**) AQP5 expression in mucous and a few intermediate cells of MEC. (**L**) Expression on acinar cell membranes of normal salivary gland tissue. A1,B,F,H1,J,K,L: original magnification ×200; A2,C,D,E,G,H2,I: original magnification ×400.

AQPs were expressed in MEC samples (Figure 1, Table 1). They were observed on cell membranes of neoplastic cells, normal salivary gland tissue, and positive controls. AQP1 was absent in the parenchymas but expressed in vascular endothelia of both MEC and normal tissue. AQP3 was expressed on the membranes of epidermoid and mucosal cells of MEC, and in myoepithelial and ductal cells of normal salivary glands. AQP5 was expressed on the membranes of mucous cells of MEC and in acinar structures of normal salivary glands.

**Figure 2:**
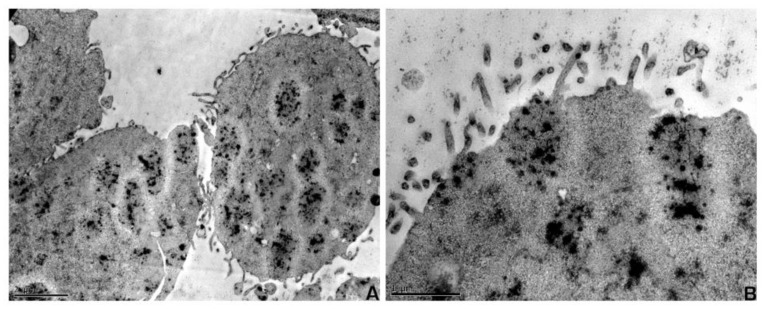
Ultrastructural aspects of UM-HMC-3A. (**A**) Epithelial cells derived from mucoepidermoid carcinoma. Note the electron dense granules in cytoplasmic vacuoles; (**B**) Mucoepidermoid carcinoma epithelial cells in culture with adhesion specializations in the cell membrane. Scale bars: A, 2 µm; B, 1 µm.

**Figure 3:**
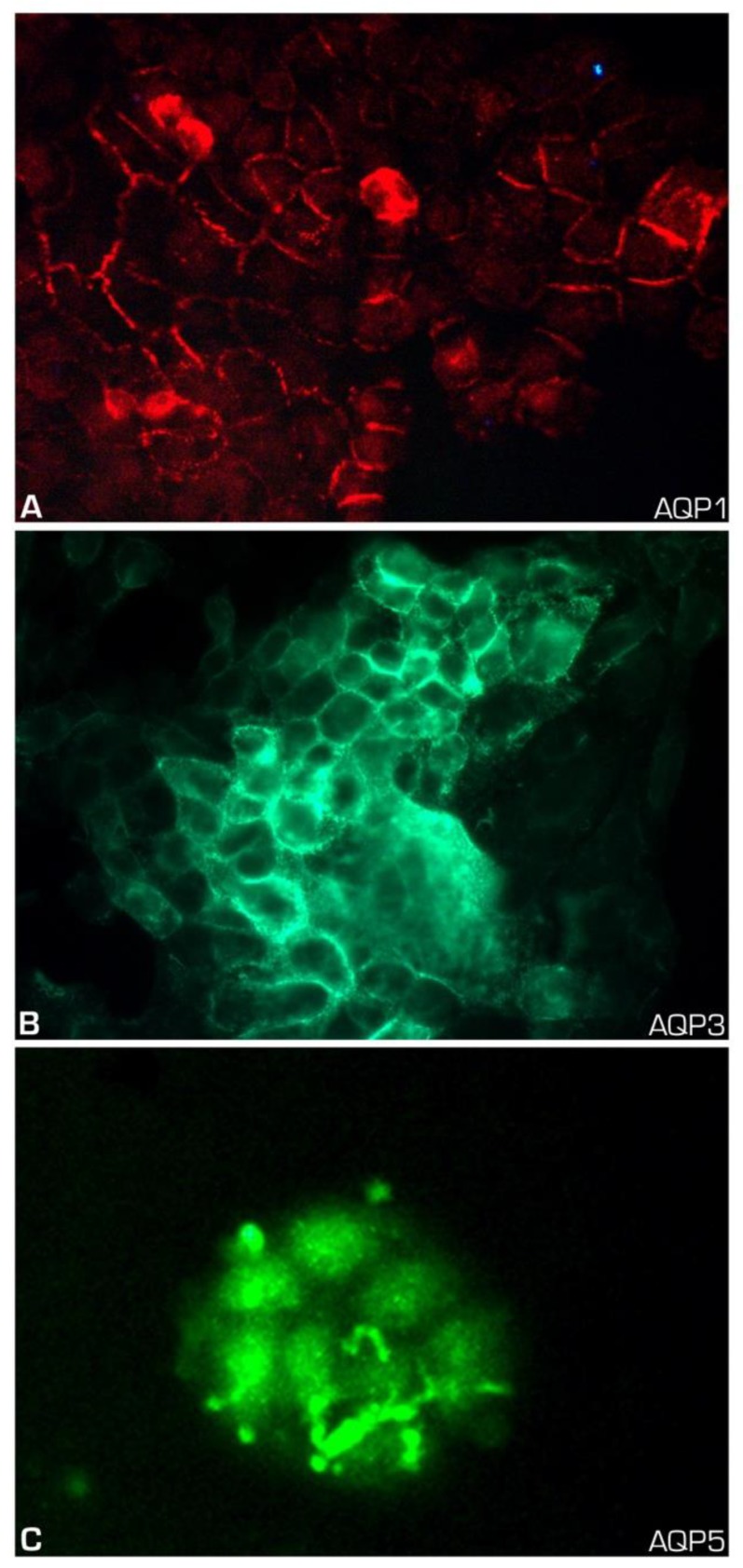
Expression of AQPs in mucoepidermoid carcinoma cell line (UM-HMC-3A). (**A**) Membrane expression of AQP1 in MEC cultured cells (original magnification ×400); (**B**) Membranous expression of AQP3 in MEC cells (original magnification ×400); (**C**) Rudimentary AQP5 expression in a few membrane foci in MEC cells (original magnification ×400).

The ultrastructural aspects of UM-HMC-3A featured epithelial cells with abundant cytoplasm, electron-dense vacuoles, and delicate cell membrane villi (Figure 2A,B). AQP expression was also evaluated in MEC cell line UM-HMC-3A, and disclosed membrane positivity for AQP1, AQP3, and, in some cells, for AQP5 (Figure 3).

**Figure 4:**
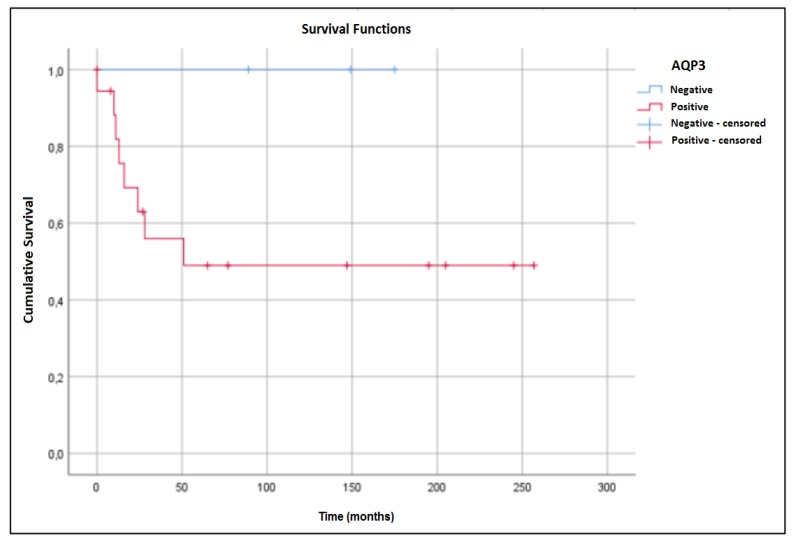
Overall survival curves of MEC patients. Five-year survival rates were 100.0% for patients without AQP3 expression and 49.0% for patients with expression (*p* = 0.153).

**Figure 5:**
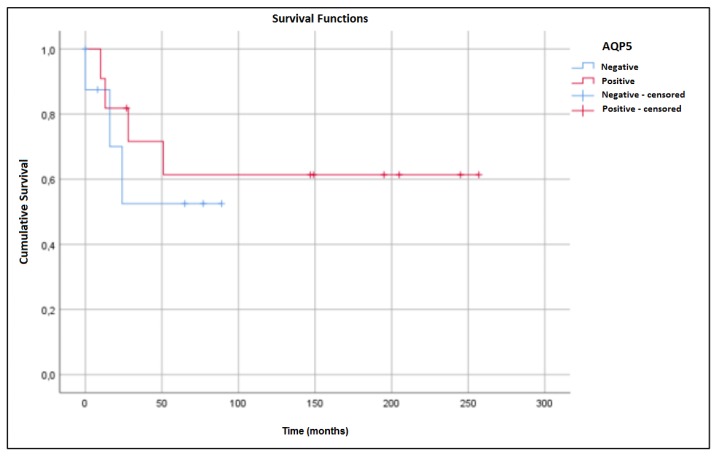
Overall survival curves of MEC patients. Five-year survival rates were 52.5% for patients without AQP5 expression and 61.4% for patients with expression (*p* = 0.574).

Statistical analysis of AQP1 expression was not performed because of its absence in MEC tumor cells. There were no significant correlations of AQP3 and AQP5 expression to demographic, clinical, or pathological characteristics. Increased five-year survival rates were observed in patients with either AQP3-negative (Figure 4) or AQP5-positive (Figure 5) tumors. However, these associations did not reach statistical significance.

## 3. Discussion

The expression profiles of APQ1, APQ3, and AQP5 in MEC tissue samples were not significantly associated with clinicopathological parameters. AQP1 was absent in the parenchymas but expressed in vascular endothelia of both MEC and normal tissue. In vitro experiments demonstrated AQP1 expression in the MEC cell line UM-HMC-3A. Delporte and Steinfeld [1] noted AQP1 expression in the rat salivary gland A5 cell line and discussed its potential role in cellular growth regulation.

De Paula et al. [24] demonstrated AQP1 expression in acini, as well as in capillaries and supposed myoepithelial cells of developing human salivary glands. In contrast, studies of adult human glands demonstrated AQP1 in the myoepithelial cells that surround acini and intercalated ducts [25,26,27]. The presence of AQP1 in vascular endothelium and myoepithelial cells suggests that its expression may be important for water transport from the vascular lumen to the salivary gland, participating in the formation of the salivary fluid [24]. AQP1 is the only AQP expressed in myoepithelial cells [25,26,27]. Because the main function of myoepithelial cells is contractile rather than secretory, it is reasoned that high water permeability enables osmotic water flow through the “basket” of myoepithelial cells into the lateral intercellular spaces of the acinus, thereby facilitating saliva production [24,25].

AQP1 may play an essential role in the interface between tumor cells and their microenvironments [11]. In MEC, AQP1 may be important for the maintenance of the vascular net, which is essential for the transport of nutrients, thereby promoting tumor growth. Ion channels and transporters have been associated with neovascularization through the mediation of endothelial cell activation, proliferation, migration, and differentiation [11,28,29]. Clapp and Escalera [30] proposed that increased vascular permeability, facilitated by AQP1, enhances water transport and initiates the angiogenic cascade. This hypothesis is further supported by the fact that AQP1 inhibition suppresses angiogenesis [31].

AQP1 overexpression has been associated with poor prognoses in breast [32], lung [33,34], colon [35], prostate [36], and pancreatic cancers [18]. In adenoid cystic carcinoma (ACC), Tan et al. [37] observed hypomethylation of the promoter region of the *AQP1* gene and overexpression of messenger RNA. Hypermethylation of the *AQP1* gene was associated with better survival rates, although *AQP1* expression was not associated with clinical outcome. In a previous study by the same group, AQP1 overexpression was demonstrated by immunohistochemistry, and strong AQP1 expression was observed in all evaluated samples. *AQP1* gene silencing inhibited tumor cell growth, suggesting that AQP1 could represent a potential therapeutic target [38].

AQP3 was identified on MEC epidermoid and mucosal cell membranes, while in histologically normal salivary glands, AQP3 was expressed in myoepithelial and ductal cells. In vitro evaluation also demonstrated AQP3 in MEC cells. De Paula et al. [24] observed AQP3 on the basolateral surfaces of acinar structures and suggested that it plays specific roles during salivary gland development and fluid secretion. AQP3 was observed in adult glands [25,26,27,39,40] and in the basolateral surfaces of acinar and ductal structures in minor salivary glands [41].

Studies have suggested that AQP3 expression might be associated with neoplastic cell migration, proliferation, and invasion [42]. Hara-Chikuma and Verkman [43] observed that AQP3 overexpression facilitates proliferation in human keratinocyte cell culture. Huang et al. [44] demonstrated that AQP3 overexpression promoted the oncogenesis of pancreatic ductal adenocarcinoma. AQP3 overexpression in esophageal squamous cell carcinoma has been associated with tumor progression and poor prognosis [45]. Matsuo and Kauano [46] demonstrated that AQP3 expression is an independent prognostic factor for lymphatic metastasis of oral squamous cell carcinoma. In this case, decreased AQP3 expression is associated with a more aggressive tumor behavior, suggesting that AQP3 is related to cellular differentiation, but not proliferation. Moreover, AQP3 overexpression has been identified in other tumors, and contributes to proliferation, epithelial–mesenchymal transition, and metastasis [13,42]. In contrast, Breyer et al. [47] demonstrated that the loss of AQP3 expression was associated with a poorer prognosis in patients with urothelial carcinoma of the bladder.

We observed that patients with AQP3-negative tumors had a better overall survival rate than patients with APQ3-positive tumors; however, the improvement was not statistically significant. Niu et al. [16] also observed AQP3 expression in MEC and other tumors of the salivary gland, although they did not investigate potential clinical associations. Ishimoto et al. [15] did not observe AQP3 expression in ACC, in contrast to Niu et al. [16], who observed AQP3 positivity in ACC. They observed that most tumors with high AQP3 expression are derived from tissues that normally express AQP3, suggesting that water metabolism through AQP3 is maintained during neoplastic transformation [16]. Consequently, AQP3 would not represent a specific indicator of benign or malignant neoplasms, thus limiting its use as a diagnostic marker.

In our study, AQP5 was expressed on the membranes of MEC mucous cells. In normal salivary glands, AQP5 was identified in acinar structures, as previously described [24,25,27,48]. In vitro evaluation also demonstrated the presence of AQP5 in MEC cells. AQP5 is one of the main AQPs of the salivary gland, playing a fundamental role in development, homeostasis, and salivary secretion [24,25]. Using an animal model, Sapmaz et al. [49] demonstrated high AQP5 expression in advanced age, suggesting a compensatory mechanism to increase salivary secretion. AQP5 expression has been associated with cellular migration, proliferation, and differentiation during neoplasia [50]. APQ5 overexpression was observed in breast ductal carcinoma and was associated with metastasis and worse prognosis [51,52], and was also associated with proliferation, migration, and metastasis in lung [53], cervical [54], ovarian [55,56], and hepatocellular cancers [57]. Similar findings have been reported in colorectal cancer [58,59,60], in which AQP5 overexpression was associated with differentiation, tumor stage, and metastasis.

In contrast, Sekine et al. [61] demonstrated that AQP5 overexpression in biliary tract carcinomas is associated with better survival rates compared to those of low expression tumors. In our study, we observed a tendency of better survival rates in patients with AQP5 expression, although this trend was not statistically significant. Ishimoto et al. [15] observed AQP5 underexpression in ACC compared to normal salivary gland. However, AQP5 overexpression was observed in tongue squamous cell carcinoma, suggesting that AQP5 has different functions in these tumors.

Some studies have demonstrated an association of AQPs with tumorigenesis. Overexpression of AQP1 and AQP5 was associated with poor prognosis in lung and esophageal cancers and soft tissue sarcomas [62,63,64]. In our study, we did not observe associations of particular aquaporins in MEC. We found no associations between AQP1, AQP3, and AQP5 and demographic, clinical, and pathological characteristics. Our results suggest that the absence of AQP3 expression and the presence of AQP5 portend better overall survival rates, although these results were not statistically significant.

We suggest that AQP expression may be involved in MEC tumorigenesis; AQP1 expression in tumor vasculature could facilitate nutrition for sustained neoplastic growth [11,30,31]. AQP3 could support tumor metabolism by enabling glycerol uptake, a key substrate of the cellular production of ATP, the essential energy source for cellular biosynthesis and, consequently, cell division and proliferation [43,50,65]. AQP5 expression in MEC could promote cellular proliferation.

Although other authors have been presenting limited results on the expression of AQPs in salivary gland neoplasms, our results add important information on their participation in MEC. In summary, our results show that, despite the fact that these molecules are important for salivary gland physiology, they may not play a distinct role in tumorigenesis in MEC. Additionally, the in vitro model may offer new possibilities to further investigate mechanisms of these molecules in tumor biology and their real significance in prognosis and possible target therapies.

## 4. Materials and Methods

### 4.1. Tissue Samples

Paraffin-embedded tissue samples from 24 MEC patients were retrieved from the archives of the Department of Pathology, A.C.Camargo Cancer Center, São Paulo, Brazil. All cases were treated at the hospital for at least 5 years. The demographic, clinical and histological details of all cases are provided in Table 2. The study was conducted in accordance with the Declaration of Helsinki and the need for informed consent was waived as the human specimens were deidentified. The protocol was approved on 19 September 2017 by the Ethics Committee of the A.C.Camargo Cancer Center (Protocol number 2433/17).

### 4.2. Immunohistochemistry

The expression of AQP1, AQP3, and AQP5 was examined in the salivary gland tissue samples. The slides were deparaffinized, rehydrated, and subjected to antigen retrieval. Details of the antigen retrieval methods and the primary antibody clones, sources, and titers are listed in Table 3. The sections were incubated in 3% aqueous hydrogen peroxide for 15 min to quench endogenous peroxidase activity, and with Protein Block (Dako, Carpinteria, CA, USA) for 30 min at room temperature to eliminate non-specific binding of subsequent reagents.

The tissue sections were incubated with the primary antibodies for 16 h at 4 °C. The antigen-antibody complexes were visualized using the Advance detection system (Dako, Carpinteria, CA, USA) and incubated with 3′3 diaminobenzidine tetrachloride (DAB) (Dako, Carpinteria, CA, USA) for 5 min. The sections were then counterstained with Mayer’s hematoxylin (Merck, Darmstadt, Germany), dehydrated, and mounted with a glass coverslip and xylene-based mounting media. Positive controls were used per the manufacturer’s instructions.

Qualitative analysis was performed using a conventional optical microscope using scores based on the percentage of stained cells and staining intensity: 0, negative staining (up to 10% of cells stained and only visible at 40×); 1, positive staining (more than 10% of cells stained and easily detectable at 10×). Additionally, the positivity was considered according to the stained structures—mucous cells, intermediate cells, squamous cells, and vasculature.

### 4.3. Cell Culture

The human salivary gland mucoepidermoid carcinoma cell line UM-HMC-3A (kindly provided by Dr. Jacques E. Nör) [66] was maintained at 37 °C in Dulbecco’s Modified Eagle Medium (DMEM; Gibco, Grand Island, NY, USA), supplemented with 10% fetal bovine serum (FBS; Eurobio, Les Ulis, France), 1% L-glutamine (Life Technologies, Grand Island, NY, USA), 1% antibiotic/antimycotic solution (10,000 units/mL of penicillin, 10,000 µg/mL of streptomycin, and 25 µg/mL of Amphotericin B) (Gibco, Grand Island, NY, USA), 20 ng/mL epidermal growth factor (EGF; Sigma-Aldrich, St. Louis, MO, USA), 400 ng/mL hydrocortisone (Sigma-Aldrich, Saint Louis, MO, USA), and 5 µg/mL insulin (Sigma-Aldrich, Saint Louis, MO, USA).

### 4.4. Immunofluorescence

UM-HMC-3A cells cultured in coverslips were fixed with 4% PFA (Sigma-Aldrich, Saint Louis, MO, USA) and washed three times in PBS pH 7.4. Permeabilization of the cells was performed with Triton X-100 1% (Sigma-Aldrich, Saint Louis, MO, USA) for AQP1 and AQP3 and 0.5% for AQP5 during 5 min at room temperature, followed by three PBS pH 7.4 washes. Cells were incubated with primary antibodies overnight at 4 °C. On the following day, after three PBS pH 7.4 washes, the cells were incubated with the secondary corresponding Alexa Fluor^®^ antibody (Alexa Fluor^®^ 594 goat anti-mouse 1:500 for AQP1 analysis, Alexa Fluor^®^ 488 goat anti-rabbit 1:500 for AQP3 analysis, and Alexa Fluor^®^ 488 goat anti-rabbit 1:200 for AQP5 analysis) at room temperature for 5 h, followed by three PBS pH 7.4 washes for 10 min. Slides were prepared with Fluoroshield Mounting Media with DAPI.

### 4.5. Electron Microscopy

The UM-HMC-3A cells were plated on a 25 cm^2^ flask at a density of 3 × 10^6^ and incubated at 37 °C in a humidified 5% CO_2_ atmosphere for 48 h. After this period, the cultured cells were scraped from the flask and centrifuged at 1000 rpm for 10 min. The pellet of cells was then fixed in 2% glutaraldehyde, washed in PBS, and post-fixed in 1% osmium tetroxide. After stepwise dehydration with ethanol (70–100%), the cells were embedded in Spurr resin, and ultrathin sectioning was performed (800–1000 Å) using an ultratome (LKB U-5 Ultratome, LKB, Sollentuna, Sweden). The cells were stained with 4% uranyl acetate and then with Reynolds lead citrate. The grids were studied and micrographed with a Philips TEM 400 transmission electron microscope, operating at 80 kV.

### 4.6. Statistical Analysis

The association between qualitative variables was evaluated by chi-squared or Fisher’s exact test as appropriate. The nonparametric Mann–Whitney or Kruskal–Wallis tests were used for the comparison of quantitative variables.

Survival curves were estimated using the Kaplan–Meier method, and the comparison between the curves was evaluated by the log-rank test. The significance level was 5% for all tests. Statistical analyses were performed using SPSS software (Version 25).

## Figures and Tables

**Table 1:** Immunoexpression of AQP1, AQP3, and AQP5 in salivary gland mucoepidermoid carcinoma parenchyma.

Protein	Positive	Negative	Number of Cases (%) *
AQP1 **	0 (0.0)	21 (100.0)	21 (100.0)
AQP3	18 (85.7)	3 (14.3)	21 (100.0)
AQP5	12 (60.0)	8 (40.0)	20 (100.0)

* Total number of cases may vary due to tissue availability for immunohistochemistry. ** AQP1 was negative in tumor parenchyma, but was present in the vasculature (intraspecimen positive control).

**Table 2:** Summary of demographic, clinical, and pathological characteristics of mucoepidermoid carcinoma patients.

Characteristic	Category	Mucoepidermoid Carcinoma (%)
Gender	Male	13 (54.0)
	Female	11 (46.0)
Age	≤40 years	08 (33.3)
	>40 years	16 (66.7)
Race	White	22 (91.7)
	Non-white	02 (8.3)
Histological grade	Low	12 (50.0)
	Intermediate	04 (16.6)
	High	07 (29.2)
	n/a *	01 (4.2)
Tumor site	Parotid	09 (37.5)
	Sublingual	01 (4.2)
	Minor Salivary Glands	14 (58.3)
Vascular invasion	Yes	03 (12.5)
	No	15 (62.5)
	n/a	06 (25.0)
Perineural infiltration	Yes	06 (25.0)
	No	13 (54.2)
	n/a	05 (20.8)
Lymph node metastasis	Yes	10 (41.7)
	No	06 (25.0)
	n/a	08 (33.3)
Local recurrence	Yes	06 (25.0)
	No	13 (54.2)
	n/a	05 (20.8)

* Information not available.

**Table 3:** Primary serum, clones, source, working titers, and antigen retrieval.

Primary Serum	Clone	Source	Working Titer	Antigen Retrieval
AQP1	1/22	Abcam	1:1000	Pressure cooker, citrate pH 6.0
AQP3	Polyclonal	Abcam	1:1000	Pressure cooker, citrate pH 6.0
AQP5	EPR3747	Abcam	1:1000	Pressure cooker, citrate pH 6.0

## Abbreviations

AQPs	Aquaporins
MEC	Mucoepidermoid carcinoma
ACC	Adenoid cystic carcinoma
